# Lumbosacral Plexopathy in Pelvic Injury – A Cause of Hip Instability in Acetabular Fractures: A Report of Two Cases

**DOI:** 10.5704/MOJ.1607.013

**Published:** 2016-07

**Authors:** N Kumar, MQ Wayne-Yap, Kwek EBK

**Affiliations:** Paras HMRI, Patna, India

**Keywords:** Pelvic injury, lumbosacral plexopathy, hip instability

## Abstract

Lumbosacral plexopathy is a rare clinical entity that results in potentially severe neurological deficit. The clinical presentation of lumbosacral plexopathy includes motor and sensory deficits. However to the best of our knowledge, hip instability secondary to lumbosacral plexopathy has not been reported in current literature. We report two cases of pelvic injury in which recurrent hip subluxation occurred following fixation of acetabular fractures. We attribute this to inadequate hip muscle tension from the associated lumbosacral plexopathy. In patients with acetabular fractures, this may lead to debilitating hip joint instability. In an already traumatized hip joint, this instability has a poor prognosis and can lead to degenerative changes with the eventual need for hip replacement.

## Introduction

Lumbosacral plexopathy is a rare clinical entity that results in potentially severe neurological deficit. The lumbosacral plexus is situated within the relative protection of the axial skeleton making pelvic trauma a possible cause of its injury. The clinical presentation of lumbosacral plexopathy includes motor and sensory deficits. However to the best of our knowledge, hip instability secondary to lumbosacral plexopathy has not been reported in current literature. We report two cases of pelvic injury in which recurrent hip subluxation followed fixation of acetabular fractures. We attribute this to inadequate hip muscle tension from the associated lumbosacral plexopathy.

## Case One

**Fig. 1:**
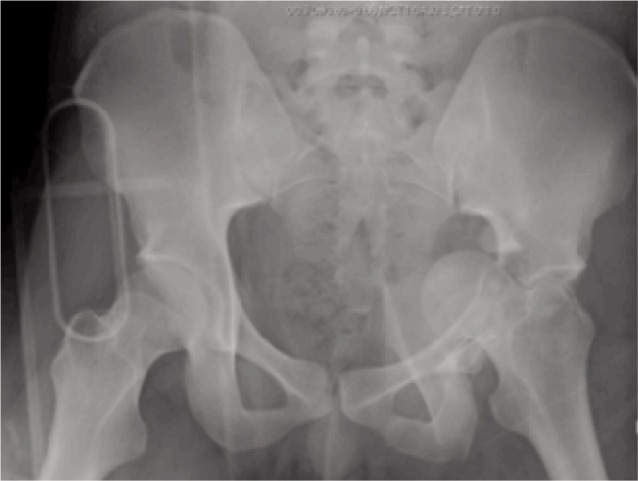
Radiograph of Case One showing T-Type Acetabular fracture.

**Fig. 2:**
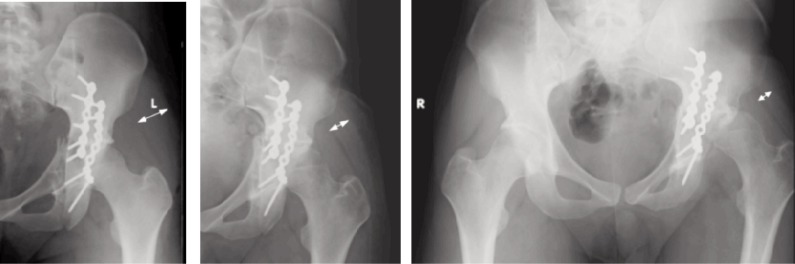
Serial radiographs of Case One after surgery, at 2 months and at 6 months, showing progressive wasting of the gluteus medius (white arrows), and eventual subluxation of the left hip.

**Fig. 3:**
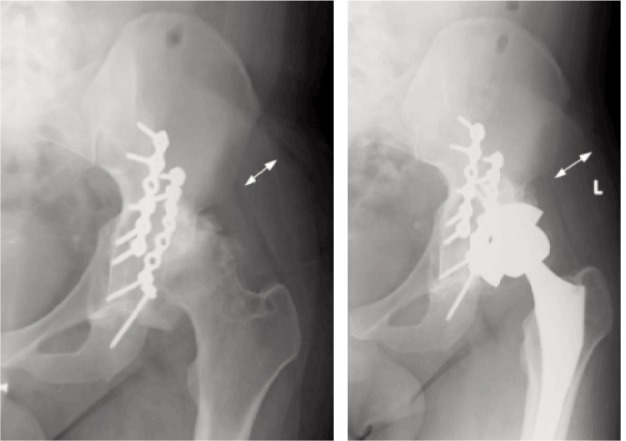
Radiograph at 2 years showing degenerative hip changes and increase in gluteus medius girth, prior to total hip arthroplasty.

A 24 years old male motorcyclist with no significant past medical history was admitted after he collided with a car. He sustained a closed T-type acetabular fracture dislocation on the left, with sacroiliac joint diastasis (Figure 1). He was also noted on presentation to have a dense ipsilateral lower limb sensorimotor deficit. He underwent immediate manipulation and reduction of the left hip with Steinman pin insertion into the distal femur for skeletal traction. Open reduction and internal fixation of left acetabulum was done semi-electively 6 days later. A posterior Kocher-Langenbeck approach was utilized with the patient in a prone position. Surgical stabilization was achieved with two posteriorly applied low profile pelvic reconstruction plates. The patient was mobilized on a wheel chair after surgery, and progressed to weight-bearing at six weeks after follow-up radiographs had shown evidence of fracture healing. In the meantime, the patient required a referral to a pain specialist for chronic neuropathic limb pain. An electromyographic study performed at six weeks after injury confirmed the presence of widespread left lumbosacral plexopathy involving the left femoral, sciatic, and gluteal nerves (Table I). He was managed supportively for the neurological injury with physiotherapy, and a walking ankle foot orthosis for his left foot drop. Unfortunately due to progressive wasting of his hip abductor muscles which was evident on serial radiographs (Figure 2), he experienced recurrent episodes of hip subluxation which were self-reducible. Radiograph showed lateral subluxation of the femoral head. The lumbosacral plexopathy continued to improve over the next two years with eventual full neurological recovery (Table II). However by this time, there was symptomatic post-traumatic osteoarthritis of the left hip and he underwent an unconstrained total hip arthroplasty at two years after the initial injury (Figure 3). No acetabular defect was seen at operation. Patient recovered uneventfully thereafter without further hip instability. At the end of one year follow-up he was walking pain-free and went back to sporting activities.

**Table I:** Showing Widespread Lumbosacral Plexopathy 6 week post trauma Case One

			**Spontane ous Activity**			**Motor Unit Potential**						
Muscle	Side	Insertion Activity	Fibs	Psw	Fasc’s	Poly	Amp	Duration	Activation	Recruit Pattern	Effort	Remarks
Tibialis Anterior	Left	Incr	1+	4+	None						Max	No Motor Unit
Gastrocnemius Medial Head	Left	Incr	1+	3+	None	Nl	Nl	Nl	Mod Reduced	Incomplete	Max	
Vastus Medialis	Left	Incr	1+	3+	None	Nl	Nl	Nl	Mod Reduced	Incomplete	Max	
Gluteus Medius	Left	Incr	1+	3+	None	Nl	Nl	Nl	Mod Reduced	Discrete	Max	
Gluteus Maximus	Left	Incr	1+	3+	None	Nl	Nl	Nl	Mod Reduced	Discrete	Max	
Semitendinosus	Left	Incr	1+	3+	None	Nl	Nl	Nl	Mod Reduced	Incomplete	Max	
Adductor Longus	Left	Nil	None	None	None	Nl	Nl	Nl	Nl	Complete	Max	

MYT- MYOTONIA, MYK- MYOKYMIA,CRD-COMPLEX REPETITIVE DISCHARGE,RFR- RAPID FIRING RATE,FF- FAST FIRING,NFR- NORMAL FIRING RATE,NL- NORMAL,PSWPOSITIVE SHARP WAVES,MUAP-MOTOR UNIT ACTION POTENTIAL, Incr - INCREASED, Decr - DECREASED, SI- SLIGHT,MOD- MODERATE,SEV- SEVERE,SCAT- SCATTERED.

**Table II:** Showing Widespread Lumbosacral Plexopathy 2 years post trauma. Case One

			**Spontane ous Activity**			**Motor Unit Potential**						
Muscle	Side	Insertion Activity	Fibs	Psw	Fasc’s	Poly	Amp	Duration	Activation	Recrut Pattern	Effort	Remarks
Tibialis Anterior	Left	Incr	2+	2+						No Muap	Sub -Max	
Extensor Hallusis Longus	Left	Incr	2+	2+						No Muap	Sub-Max	
Gastrocnemius												
Lateral Head	Left	Incr	1+				Nl/+1	Long		Incomplete	Sub- Max	
Peroneus Longus	Left	Incr	1+				Nl/+1	Long		Incomplete	Sub-Max	
Tibialis Posterior	Left	Incr	1+				Nl/+1			Discrete	Sub- Max	Tech
Difficult												
Semitendinosus	Left	Incr					Nl/+1	Long		Incomplete	Sub-Max	
Vastus Medialis	Left						Nl/+1	Si Long		Complete	Sub-Max	
Vastus Lateralis	Left						Nl/+1	Si Long		Incomplete	Sub-Max	
Iliopsoas	Left	Nl	None	None	None	Nl	Nl	Nl	Nl	Complete	Max	

MYT- MYOTONIA, MYK- MYOKYMIA,CRD-COMPLEX REPETITIVE DISCHARGE,RFR- RAPID FIRING RATE,FF- FAST FIRING,NFR- NORMAL FIRING RATE,NL- NO RMAL,PSW- POSITIVE SHARP WAVES,MUAP-MOTOR UNIT ACTION POTENTIAL,Incr- INCREASED, Decr- DECREASED, SI- SLIGHT,MOD- MODERATE,SEV- SEVERE,SCAT- SCATTERED.

## Case Two

**Fig. 4:**
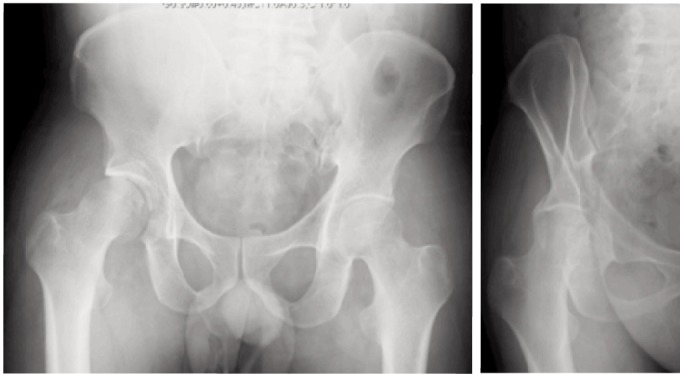
Initial radiographs of Case Two showing a Transverse-posterior wall fracture of the right acetabulum.

**Fig. 5:**
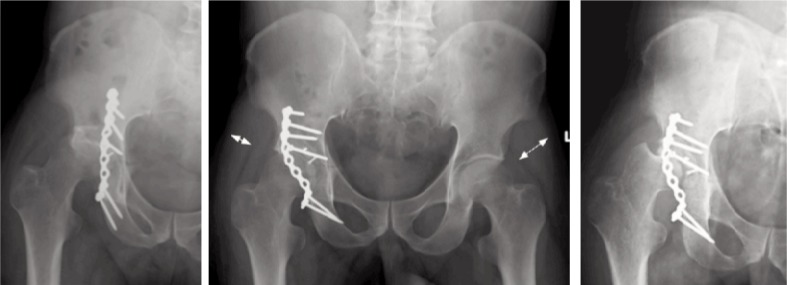
Radiographs at one month showing lateral subluxation, after revision surgery, and at 5 months showing persisting subluxation. Note the wasting of the gluteus medius (white arrow) compared to the contralateral hip (dotted arrow).

A 44 years old cargo mover with no past medical history of note suffered a closed crush injury to his right hip from work. He sustained a transverse-posterior wall fracture of the right acetabulum (Figure 4) and underwent open reduction and internal fixation four days later via a posterior Kocher-Langenbeck approach. Posterior wall marginal impaction was addressed with trochanteric bone graft and headless compression screws. The posterior wall was fixed with lag screws and the column fractures were buttressed with a single posterior low profile pelvic reconstruction plate. Final reduction was stable. The post-operative recovery was uneventful except for complaints of right sided neuropathic pain with patchy sensory loss. The patient was placed on non-weight-bearing exercises and discharged but presented one month later with right lower limb numbness and instability in the hip joint. Radiographs showed a displaced supero lateral posterior fragment with subluxation of the joint. The patient underwent revision of his acetabular fixation and was noted intra-operatively to have loosened implants without evidence of infection. The lag screw and plate were revised and was again found to be stable after reduction. On follow-up the patient continued to have right lower limb neuropathic pain and developed gross wasting of the lower limb musculature. He also complained of recurrent subluxation of his hip joint which he was able to reduce himself (Figure 5). An electromyographic study performed at six weeks showed evidence of right lumbosacral plexopathy, affecting the sciatic, gluteal and peroneal distribution. The right acetabular fracture united eventually and the patient was counselled for total hip arthroplasty after the plexopathy recovers.

## Discussion

Lumbosacral plexopathy is a well-described clinical entity in pelvic trauma^1^. Huittinen performed autopsies of 42 victims of lethal trauma and pelvic fractures and established that 20 bodies (48%) had sustained damage to the lumbosacral plexus^2^. However incidence amongst survivors is much lower. Kutsy *et al*^3^ concluded that 0.7% of almost 3000 patients with pelvic and acetabular fractures developed lumbosacral plexopathy. Other authors have reported 0.5-2.27% incidence of lumbosacral plexopathy^4^. The proximity of the lumbosacral plexus to the sacral bone and sacroiliac joint would suggest a higher incidence of lumbosacral plexopathy with injury to these structures, with the extent of nerve injury being proportionate to the severity of the posterior pelvic injury. Majeed suggested that nerve recovery starts three months after injury and stops after two years^5^. Weis evaluated that 11 of 28 patients have electromyographic changes due to lumbosacral plexus injury. The EMG changes were diffuse and the resulting neurological deficit interfered with the rehabilitation process.

The anterior division of lumbar, sacral and coccygeal nerves form the lumbosacral plexus. The common causes of lumbosacral plexopathy are direct compression, pelvic injury, diabetic neuropathy and parturition. They frequently present with anterior thigh pain and proximal muscle weakness. The muscle weakness is pronounced in the quadriceps, glutei and hamstrings. These may lead to gross wasting and a lurching gait. Weakness of abductors and Iliopsoas muscles lead to instability of the hip joint.

To our knowledge no case of recurrent hip subluxation has been reported due to lumbosacral plexopathy, much less in patients who have undergone acetabular fracture reconstruction. Apart from lumbosacral plexopathy other causes of recurrent hip instability include improper and poor fixation, disuse atrophy and congenital laxity.In both of our cases there was good bony apposition and union which was confirmed by CT scan. In the first case we felt it prudent to wait for resolution of the plexopathy before proceeding with total hip arthroplasty. This reduced the risk of subluxation of the hip replacement from the weakened abductor musculature. The second case illustrated the variable presentation of the lumbosacral plexopathy, which was not suspected as the underlying pathology for instability until after the revision fixation and nerve conduction study results became available.

Both these cases, however, illustrate the poor prognosis associated with this combination of neurological and bony injury, with the inevitable development of post-traumatic hip joint arthropathy, and the need for eventual hip replacement surgery.

## Conclusion

Patients with acetabular fractures and pelvic injury may have varying degrees of lumbosacral plexopathy. In patients with acetabular fractures, this may lead to debilitating hip joint instability. In an already traumatized hip joint, this instability has a poor prognosis and can lead to degenerative changes with the eventual need for hip replacement. Careful planning for surgery after complete resolution of the neurological injury is advised to reduce post-arthroplasty complications.
